# Simultaneous Production of Biogas and Electricity from Anaerobic Digestion of Pine Needles: Sustainable Energy and Waste Management

**DOI:** 10.3390/biotech13030035

**Published:** 2024-09-05

**Authors:** Deepak Sharma, Rishi Mahajan, Vikas Baghel, Saurabh Bansal, Vishal Ahuja, Gunjan Goel

**Affiliations:** 1 Department of Biotechnology and Bioinformatics, Jaypee University of Information Technology Waknaghat, Solan 173234, Himachal Pradesh, India; deepaksharma2899@gmail.com (D.S.); rishisml@gmail.com (R.M.); saurab.bansal02@gmail.com (S.B.); 2 Department of Biotechnology, Chandigarh College of Technology, Chandigarh Group of Colleges Landran, Mohali 140307, Punjab, India; 3 Department of Microbiology, Chaudhary Sarvan Kumar Krishi Vishwavidyalaya, Palampur, Kangra 176061, Himachal Pradesh, India; 4 Department of Electronics and Communication Engineering, Jaypee University of Information Technology Waknaghat, Solan 173234, Himachal Pradesh, India; vikas.baghel@juit.ac.in; 5 University Institute of Biotechnology, Chandigarh University, Mohali 140413, Punjab, India; 6 University Centre for Research and Development, Chandigarh University, Mohali 140413, Punjab, India; 7 Department of Microbiology, Central University of Haryana, Mahendragarh 123031, Haryana, India

**Keywords:** anaerobic digestion, direct interspecies electron transfer, pine needles, conducting materials

## Abstract

Power scarcity and pollution can be overcome with the use of green energy forms like ethanol, biogas, electricity, hydrogen, etc., especially energy produced from renewable and industrial feedstocks. In hilly areas, pine needles are the most abundant biomass that has a low possibility of valorization due to high lignin content. On the other hand, anaerobic digestion (AD) of lignin and animal waste has low biogas yield due to poor conductivity. This study focuses on the simultaneous production of biogas and electricity through the co-digestion of cow dung and pine needles. The digester was initially established and stabilized in the lab to ensure a continuous supply of inoculum throughout the experiment. The optimization process involved the determination of an ideal cow dung-to-water ratio and selecting the appropriate conductive material that can enhance the energy generation from the feedstock. Afterward, both batch and continuous anaerobic digestion experiments were conducted. The results revealed that the addition of powdered graphite (5 mM), activated charcoal (15 mM), and biochar (25 mM) exhibited maximum voltage of 0.71 ± 0.013 V, 0.56 ± 0.013 V, and 0.49 ± 0.011 V on the 30th, 25th and 20th day of AD, respectively. The batch experiment showed that 5 mM graphite powder enhanced electron transfer in the AD process and generated a voltage of 0.77 ± 0.014 V on the 30th day, indicating an increase of ~1.5-fold as compared to the control (0.56 ± 0.019 V). The results from the continuous AD process showed that the digester with cow dung, pine needle, and a conductive material in combination exhibited the maximum voltage of 0.76 ± 0.012 V on the 21st day of AD, while the digester with cow dung only exhibited a maximum voltage of 0.62 ± 0.015 V on the 22nd day of AD, representing a 1.3-fold increase over the control. Furthermore, the current work used discarded plastic items and electrodes from spent batteries to emphasize waste management and aid in attaining sustainable energy and development goals.

## 1. Introduction

Power shortage is one of the greatest challenges in the current scenario, which may be due to several reasons like environmental and climatic disturbances such as droughts and storms, excessive consumption, and wastage. Power shortages in some of the territories like Brazil, China, India, the United States, and many European countries are mainly due to overcrowded populations, energy and fuel wastage, and civil and international wars. Besides normal living systems, power shortage also has long-term as well as short-term negative impacts on commercial firms, production houses, revenue generation, and economic growth due to reliance on electricity. As per the World Bank’s report, electricity supply has been identified as a major constraint on business activities, resulting in a loss of around USD 82 billion per annum in developing countries [1]. An alternate source of energy, or more precisely electricity, may not be sufficient alone, but it would contribute significantly to overcoming the shortage. Different thermal, chemical, and microbial conversion approaches have been explored for cost-effective energy generation along with waste management (Table 1).

The microbial potential of electricity or voltage generation is an interesting fact that was discovered over a century ago [7,8], but in the past few decades, microbial electricity has received attention around the world [9,10]. Microbial fuel cells (MFCs) are a unique technology that involves electricity generation by using the potential of microorganisms. It involves the biological oxidation of organic matter and the subsequent transfer of electrons to the anode. The utilization of waste materials as substrates in MFCs is a sustainable approach to energy production [11].

In recent years, the MFC approach has been used with anaerobic digestion and waste management [12,13,14]. The concept has been widely exploited for power generation from sugar industry effluent by Sreelekshmy et al. [15], food waste by Yoshizu et al. [16], methane from polylactic acid by Liu et al. [17], ethanol from kitchen waste by Sondhi et al. [18], and electricity from chicken feather by El Salamony et al. [19]. The anaerobic digestion in bioreactors generates chemical energy in the form of methane, which can be used as a fuel directly or for the subsequent production of electricity. It is reported that bacteria can transfer more than 80% of the electrons present in organic substrates to generate electricity in a typical MFC [20]. In MFCs, the anodic potential is directly proportional to the free energy of the substrate. Apart from electricity, MFCs can also produce hydrogen by utilizing organic compounds such as acetate [21,22]. The microbial diversity in MFCs is influenced by the type of substrate and environmental conditions. In MFCs, substrate utilization is considered a crucial biological factor that plays an important role in electricity generation [23,24]. There is a wide range of substrates used in MFCs with the ultimate goal of pollutant elimination, the transformation of waste biomass, and the production of sustainable energy, making this approach a good economic asset for industries [25].

Lignocellulosic materials are among the largest biomass resources across the globe [26]. The amount and availability of these lignocellulosic biomass materials from agricultural, forest, and industrial sources advocate their utilization as an economic substrate for sustainable energy production [27,28]. However, their complex and inert structure limits the direct availability of nutrients for microorganisms as substrates, as well as the production of value-added products like biogas. Chir pine (*Pinus roxburghii*) is the dominant plant species in the Himalayan subtropical pine forests of Himachal Pradesh [29,30]. It is estimated that the pine litterfall occupies 2–3 ha^−1^ year^−1^ on forest floors [29,31]. Naturally, the degradation of pine needles is very slow due to the greater concentration of lignin in its biomass [32]. Pine needles contain around 20–25% lignin, along with cellulose and a fraction of hemicellulose. The higher concentration of lignin makes biodegradation slower and difficult, which is mainly responsible for the higher accumulation of leaves on the forest floor. This leads to the generation of mats on forest floors and thus presents a primary risk of forest fires annually in India [33,34,35]. The burning of pine needles in forests leads to the generation of harmful gases and hazardous volatile compounds [36]. The higher lignin content of pine needles is also a challenge for their utilization in anaerobic digestion. In addition, pretreatments of pine needles led to a significant reduction in lignin content, but the addition of pine litter as a substrate in anaerobic digestion decreased its efficiency regardless of the inoculum size [37], mainly due to poor conductivity and electron transfer rate, which are attributed to indirect interspecies electron transfer (IIET). This issue can be countered by using conducting material that increases the electron transfer via direct interspecies electron transfer (DIET) [38,39]. Conductive materials play a significant role in enhancing AD processes. Their inclusion in anaerobic digesters impacts the microbial activity and efficiency of organic waste breakdown, especially in the context of bioelectrochemical systems. Hence, in the last few years, scientists have emphasized the use of conductive materials to enhance power generation in MFCs. It has been suggested that DIET mechanisms increase methane production by 16–60% [38]. In an investigation using an MFC to analyze the effect of magnetic nanoparticles on anaerobic digestion, the magnetite nanoparticles and magnet digester achieved the highest biogas production of 545.2 mL/g_VSfed_, which was significantly higher than the 117.7 mL/g_VSfed_ produced by the control [40].

Similarly, comprehensive research investigated the effect of adding 1–5 g/L granular activated carbon during the AD of 0.5–4 g/L 5-hydroxymethylfurfural. The addition of 5 g/L GAC resulted in the highest increase, enhancing the sludge’s electron transfer activity by 27.0–75.0% and the peak methane generation rate by 27.5–68.1% when compared to the control group [41]. The use of activated carbon for energy production has gained much popularity due to enhancing the electron transfer between the cathode and microorganisms in MFCs [42,43,44,45].

Considering all the issues and challenges, the present work is targeted toward the use of different carbon forms (activated charcoal powder, graphite powder, and biochar powder) as a conductive material to enhance the electron transfer in the AD of pine needles along with the generation of electric potential.

## 2. Materials and Methods

### 2.1. Materials

Fabricated glass digester, silicon glue, and silicon tubes were purchased from JSGW, Ambala, Haryana, India. Fallen and dried pine needles (litter) were collected from the forests of Kandaghat region (30.9702° N, 77.1054° E), Solan, India, and cleaned properly. The leaves were dried to reduce the moisture content below 5 ± 0.5% and were ground using an electric grinder to produce its powder for further use. Graphite powder was purchased from M.P. Biomedicals (purity > 99%), India. Activated charcoal powder was bought from Merck India (purity > 99%), whereas biochar was purchased from the local market and was ground to make powder (used with its native purity). Arduino and jumping wires were from purchased online platforms.

### 2.2. Experimental Setup and Stability of an Anaerobic Digester

The lab scale anaerobic digester (0.75 L working capacity) was established using an anaerobic digest from an ongoing biogas plant (Jaunaji, Solan, H.P., India). The digester was maintained under mesophilic conditions at 35 °C and selected for optimal year-round performance based on environmental conditions. Its stability was assessed through the total gas production and methane yield, measured using a methane analyzer (HNL Systems Private Limited, Mumbai, India). The digester was continuously fed with fresh cow dung (obtained from a nearby village) at a regular interval of 3 days. The digester was used as a source of inoculum in the current study.

### 2.3. Fabrication of MFC Systems

Briefly, 500 mL wash bottles (plastic) were used to prepare digesters with a working volume of 400 mL. The graphite rods from the waste batteries were used to make the anode, and stainless-steel jumping wires were used to make the cathode (purchased from the local market). Silicon tubes (purchased from the local market) were used for the outlet, inlet, and gas portals in the digesters and were fixed firmly with silicon glue.

#### 2.3.1. MFC Operation

For the operation, pine needle was used in varying concentrations (1–5%) of the feed volume of the digester. Along with pine needle, cow dung was also added in different cow dung–water ratios (1:1, 1:2, and 2:3), and the current was measured using a DC Voltage (V) Rish 14S multimeter for 30 days at regular intervals of 5 days. Experiments were performed in triplicate. MFCs in Test 1 were fed with cow dung slurry and pine needle powder; in Test 2 with cow dung slurry, pine needle powder, and a conductive substance; and in the control with simply cow dung slurry.

#### 2.3.2. Selection of Conductive Materials

Conductive materials like graphite powder (MP Biomedicals India Pvt Ltd., Navi Mumbai India), activated charcoal powder (Merck, Mumbai India), and biochar powder (from the local market) were screened based on prior studies in the field. All three had varied concentrations of 0–30 mM and the DC was measured for 30 days with a regular interval of 5 days. Experiments were performed in triplicate.

### 2.4. Batch Experiment of MFC

The batch experiment was performed using optimized feed (cow dung–water) and substrate (pine needle) concentration. The experiment was conducted at 35 °C for a duration of 40 days at a 400 mL scale. Graphite powder (5 mM optimized) was used as conductive material. The experiment was carried out along with its control (Table 2). All the systems were run in triplicate. Voltage (V) was measured for each system using a Rish 14S multimeter after a regular interval of 5 days, whereas methane was also measured using a portable methane analyzer. For comparison, all sets were run in parallel and at least in triplicate, which also ensured replicability.

### 2.5. Scale-Up Studies and Continuous Operation

The scale-up studies involved three steps, i.e., (1) fabricating fermenters for scale-up studies, (2) designing circuits for continuous monitoring and programming, and (3) monitoring the current in continuous mode for 40 days using Arduino.

The indigenous model of fermenters was developed with waste plastic containers of 5 L capacity. The feeding inlet, outlet, sampling port, anode, and cathode were the basic required features and were made with the easily available waste resources. The anode consisted of a graphite rod, while a stainless-steel wire was used as a cathode. For the continuous monitoring of electrochemical potential, the circuit was designed with the help of experts from the Electronics Department of the university, followed by programming using Arduino (purchased from the online store) with MATLAB R2016a. The Arduino software (Arduino IDE 1.8.5) was installed in the computer from its official website https://www.arduino.cc (accessed on 30 June 2018) and the whole system was interconnected to each other.

This continuous operation model was designed for scale-up studies using a previously optimized conductive material, i.e., graphite powder (5 mM), pine needle as the substrate (1%), a cow dung–water ratio of 2:3, and a temperature of 35 °C for 40 days at 3 L working capacity. The details of the digesters’ setup are listed in Table 3. Experiments were performed in triplicate.

### 2.6. Statistical Analysis of Batch and Continuous Models

The batch and continuous fermentation trials were conducted in triplicate, and the mean values were used for analysis. The statistical analysis of both models was conducted by two-way ANOVA and correlation with MS Excel loaded with a data analysis toolkit.

### 2.7. Economic Feasibility and Cost Analysis

The process economics was evaluated at the lab scale as well as the bench scale trial for biogas production. The cost was also estimated for digester fabrication with all the materials, transport, and storage. The operational cost was calculated separately with the materials required to run, their processing, and output. The material and energy cost was calculated by considering a commercial price.

## 3. Results

### 3.1. Establishment of Digester and Its Stability

**Figure 1:**
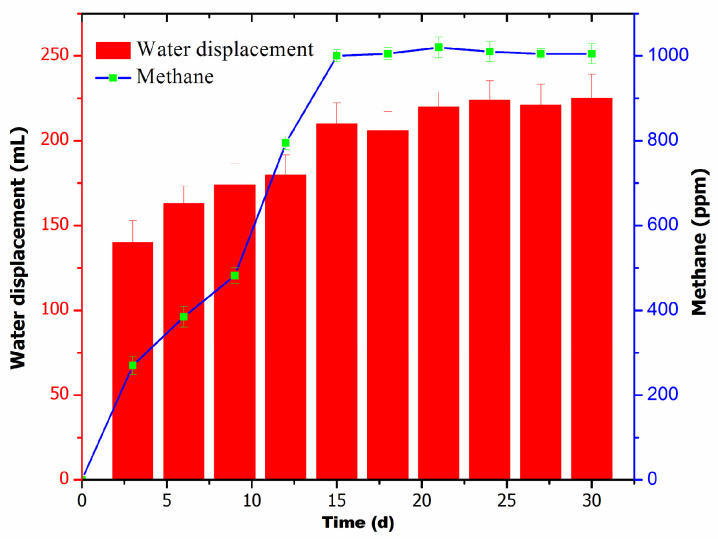
Digester stability in terms of water displacement by biogas and methane production.

The main objective of the current experiment was to establish a stable lab-scale digester that can be used as an inoculum source in the current study. A constant digester source offers benefits like a uniform microbial community and their load. The established digester was assessed for its stability in terms of the total volume of water displaced by gas and the generation of methane. A stable volume of gas production was achieved from 15 days onward (Figure 1).

A digester in the laboratory was assessed for its stability based on the total biogas and methane production. Figure 1 illustrates that the maximum water displacement (approximately 210 mL) and methane production (1000 ppm) were observed after the 15th day, peaking until the 30th day. The results from methane gas production also suggested that the digester achieved its stability after 15 days. It is clearly depicted that in the digester, methane production was low initially, and it increased gradually day by day. However, the digester started methane production >1000 ppm after 15 days, which was constant throughout the experiment. Following the stabilization of the digester, further optimization, batch, and continuous studies were conducted.

### 3.2. Optimization of Various Parameters

**Figure 2:**
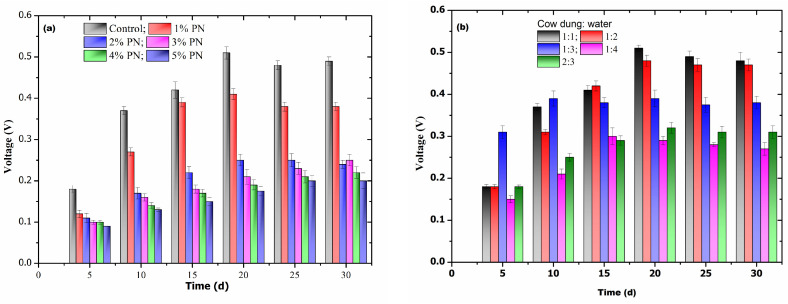
(**a**) The effect of various pine needle concentrations on the AD process, and (**b**) the optimization of various cow dung-to-water ratios for feeding.

Considering the effect of substrate concentration and cow dung-to-water ratio on the AD process, the mixture feed using pine needles and cow dung proved effective in electricity generation. As shown in Figure 2a, cow dung alone (control) exhibited a maximum voltage of 0.5 ± 0.01 V on the 20th day of digestion. After the 20th day, there was a slight decrease in V, but it was constant throughout the digestion process. It is also clearly indicated that the V decreased as the concentration of pine needles increased from 1% to 5%, and hence 1% pine needles was selected as the optimum concentration, as it led to minimum hindrance during the digestion process. Figure 2b shows that the 1:1 cow dung-to-water ratio as feed was the optimum ratio, which yielded a maximum V of 0.52 ± 0.015 V on the 20th day. A further increase in the water content had a negative effect on the process yield, and a cow dung-to-water ratio of 1:4 resulted in a minimum V throughout the experiment.

Notably, 1% pine needle was found to be the best concentration for maximum product output and substrate digestion. At a lower concentration of pine needles in the feed, there was no effective change in voltage or biogas production, while a further increase in pine needles resulted in reduced energy yield, which might be due to the inhibitory effect of lignin, which hinders microbial metabolism.

### 3.3. Selection of Conductive Materials and Their Concentrations for the AD Process

**Figure 3:**
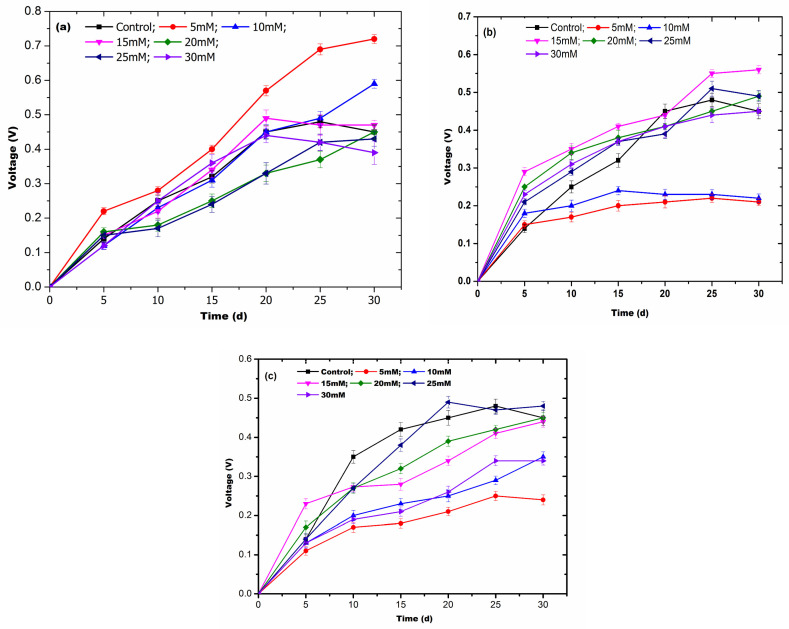
The effect of varying concentrations of conductive materials, namely (**a**) graphite powder, (**b**) activated charcoal powder, and (**c**) biochar powder, on the AD of pine needles to produce current.

In the present study, three carbon-based conductive materials (activated graphite powder, activated charcoal powder, and biochar powder) were used to enhance the DIET-based AD of pine needles and V production. The addition of conducting materials had a conducive effect on V generation, and 5 mM graphite powder exhibited 0.71 V ± 0.013 V on the 30th day (Figure 3a), while 15 mM activated charcoal powder generated a maximum of 0.56 V ± 0.013 on the 25th day of AD (Figure 3b). Moreover, biochar powder exhibited a maximum of 0.49 V ± 0.011 on the 20th day of AD (Figure 3c) but was significantly low as compared to the control system (without pine needles). From the comparative analysis of all three conductive materials, graphite powder (5 mM) was found to be most suitable for the AD of the lignocellulosic substrate and the subsequent production of maximum current.

### 3.4. Batch Experiment

**Figure 4:**
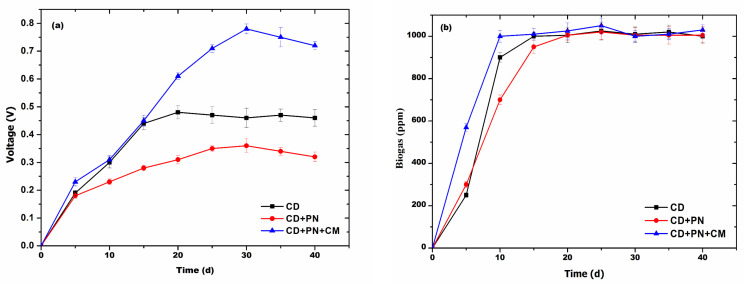
The effect of graphite powder (5 mM) on the batch AD process of pine needles and the generation of (**a**) voltage and (**b**) biogas.

The batch experiment showed that 5 mM graphite powder enhanced the electron transfer in the AD process and resulted in 0.77 ± 0.014 V on the 30th day, indicating an increase of ~1.5-fold as compared to the control (0.56 ± 0.019 V) (Figure 4a). The methane production during the AD process was found to be >1000 ppm after the 10th day in the system with only cow dung, while the system with cow dung in combination with pine needles was found to produce methane above 1000 ppm after the 20th day. Moreover, the system with cow dung and pine needles in combination with a conductive material was found to exhibit a methane concentration of >1000 ppm from the 10th day onwards. In all the systems during the initial phase, both the current and methane production were low, which may be due to the lag period stabilization of digesters. Moreover, the voltage of the system and methane production slowly started decreasing after 30th days in the digesters with cow dung with pine needles, whereas this decline in the other two systems was found after the 35th day, which may be either due to the deficiency of the substrate in the system or due to the accumulation of ammonia and other substances.

### 3.5. Continue Scale-Up Studies

**Figure 5:**
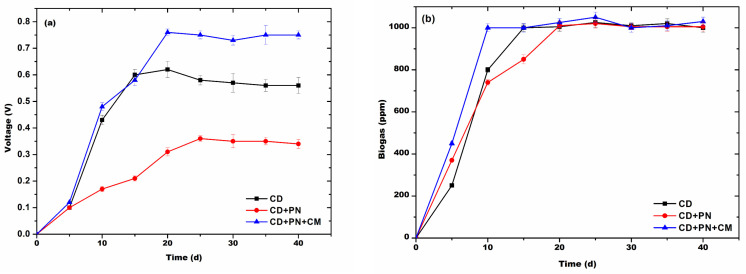
The effect of graphite powder (5 mM) on the continuous AD process of pine needles and the generation of (**a**) voltage and (**b**) biogas.

The results from the continuous AD process showed that the digester having cow dung, pine needle, and the conductive material in combination exhibited a maximum voltage of 0.76 ± 0.012 V on the 21st day of AD, while the digester with only cow dung exhibited a maximum voltage of 0.62 ± 0.015 V on the 22nd day of AD (Figure 5a) and was almost the same in the remaining period of the AD process. The digester with cow dung in combination with pine needles exhibited a remarkably low current throughout the digestion, with a maximum of 0.36 ± 0.012 V on the 24th day of AD (Figure 5a), and it was nearly unchanged in the remaining period of the AD process. Methane production was observed to be >1000 ppm for the digester with cow dung only and the digester with cow dung, needle, and conductive materials on the 15th day and the 10th day of AD, respectively, and it was unchanged in the remaining period of the AD process. The digester with cow dung exhibited a methane concentration >1000 ppm on the 20th day of AD and was constant thereafter. It was observed that up to the 6th day, the current–voltage and methane production was low in the digesters; this may be due to the initial stabilization of microbial communities in the digesters.

The co-substrate digestion of cow dung and pine needles had a higher biogas yield in comparison to cow dung slurry only, which was further improved by the addition of conducting materials like graphite. The main outcome of previous research suggested that C/N has a prominent role in biogas production and anaerobic digestion. In the current work, the addition of graphite and pine needles possibly maintained the C/N ratio of feedstock along with improving the buffer capacity and electron transfer rate of the system. Some of the reports have also emphasized that carbon provides habitat to microflora and improves microbial activity. This work has shown the possible valorization of waste biomass like cow dung and pine needles for energy generation. The residues generated after digestion can also be used as biofertilizers.

Both models were evaluated for model fitness and suitability for consideration. ANOVA analysis revealed that the ‘*p*-values’ of the batch model and the continuous model were 0.0004 and 0.00037. For model fitness, the *p*-value must be below 0.05, and both batch and continuous models had lower than 0.05, which evidently suggests that the model is fit for consideration. In addition, correlation analysis also revealed that biogas, as well as voltage generation, had a high correlation with digestion time, at a significance level of 0.001.

### 3.6. Economic Feasibility Analysis

The experimental trials were run with cow dung as the main substrate, pine needles as the co-substrate, and graphite powder as the conducting material. Therefore, a comparative assessment was conducted with cow dung, pine needle, and graphite powder (set 1); cow dung and pine needle (set 2); and cow dung (set 3). At the bench scale, the fabrication cost was INR 21.76 per digester (USD 0.26), while the operational cost was INR 56.59 per digester (USD 0.68) including a power requirement of 180 units. The digester operation resulted in a maximum voltage of 0.77 V on the 30th day and a biogas yield of more than 706.3 mg.m^3^ on the 7th day, while in sets 2 and 3, the time for similar biogas yields was the 25th day and the 12th day. Similarly, the maximum voltage was 0.3 V in set 2 and 0.45 V in set 3.

Additionally, in the bench scale trial, the fabrication cost per digester was INR 92.17 (USD 1.11), and the operational cost was INR 75.17 (USD 0.90). The variation in voltage exhibited a similar pattern, as the maximum voltage rates in sets 1, 2, and 3 were 0.76 V (21st day), 0.36 V (24th day), and 0.62 V (22nd day). Also, in biogas, the maximum yield was on the 10th day (set 1), the 20th day (set 2), and the 15th day (set 3). The greatest highlight of this work was the use of plastic containers; forest litter; and electronic waste like jerry cans, plastic bottles, pine needles, and graphite rods. These waste materials reduced the fabrication cost of the digester by approximately 40% (lab scale) and 68% (bench scale).

## 4. Discussion

Agricultural biomass has high potential due to the high concentration of organic nutrients like carbohydrates, proteins, and minerals. Previous works have revealed that the microbial digestion of waste materials can be cost-effective in fuel production [46,47]. The current work also explored the potential of cow dung and pine needles as a potential feed for biogas and electricity generation. Previous researchers have established that the suitable hydraulic retention time (HRT) ranges from 30 to 40 days for mesophilic anaerobic digesters [48]. Faria [49] employed reactors with HRT values ranging from 7.5 to 16 days and organic volumetric loads of 20 to 35 g COD/L.d for methane production. However, the average HRT varies depending on the digester and the type of substrate used in digestion, spanning from 2 to 30 days [50]. The low production of methane in the initial 3–6 days can be considered a lag period of microbial communities present in the digester [51]. The major hindrance in anaerobic digestion is the transfer of electrons. The addition of conductive materials has been previously well recognized to enhance the AD process through the DIET mechanism from various substrates to produce methane and other products [52,53]. In the DIET mechanism, electrons are directly transferred between microorganisms that oxidize volatile fatty acids and methanogens, minimizing electron losses [54,55]. Ruan et al. [56] enhanced the performance of an anaerobic reactor by adding scrap iron, resulting in a 1.5-fold improvement in chemical oxygen demand removal efficiency and a 1.4-fold increase in methane yield. In addition to these, multiple investigations have shown that the development of DIET mechanisms and the inclusion of conductive materials, such as granular activated carbon and semi-conductive iron oxide minerals, resulted in enhanced methane generation [55,57,58]. Graphite rods as conductive material could be utilized as a component of digesters because of their excellent surface, which is favorable for the growth of current-generating biofilms in MFCs [52,59,60]. Furthermore, previous reports have suggested that graphite plays a vital role in the transfer of electrons in the AD process [61,62] and can substitute PilA or OmcS for electron transfer [63]. In the current work, it was observed that higher concentrations of pine needles impeded the anaerobic digestion process due to the presence of inaccessible lignin. To address this challenge, various conductive materials were tested to enhance anaerobic digestion and facilitate simultaneous electricity production.

The batch experimentation using MFCs showed that the system with iron–graphite rods increased biogas production by 22.4% and led to an 11% increase in the VSS removal under the applied voltage of 0.3 V [64]. MFCs for the dye decolorization of acid red 27 were reported to produce a power density of 940.3 ± 4.2 mW m^−2^ in the presence of clinoptilolite fine powder-coated graphite felt bioanode, which was greater than the system with bare graphite felt bioanode (458.8 ± 5.0 mW m^−2^) [65]. In another study, conductive Fe_3_O_4_ was suggested to support the DIET between *Syntrophomonas* and *Methanosaeta* for accelerating the anaerobic digestion of waste sludge [66]. The DIET via graphene was reported to enhance methane yield by 25% and its production rate by 20% in the AD of ethanol [61]. In an experiment on electrical conductivity, when graphite felt was added to a semi-continuous reactor, the DIET was enhanced for the degradation of propionate and butyrate to yield CH_4_. The results from the study showed that CH_4_ production and the production rate increased by 19.1% and 16.7%, respectively. Furthermore, the microbial community analysis of the digester revealed that the relative abundance of *Geobacter*, *Methanosaeta*, and *Methanosarcina* species was greatly improved in the presence of graphite felt, which could participate in the DIET [54]. Conductive carbon cloth was observed to be very effective in stimulating the syntrophic conversion of ethanol to methane in mixed cultures. Moreover, it could readily be incorporated as part of the anaerobic digester design to provide a permanent conductive conduit for syntrophic metabolism [67]. Considering the available literature, the current work showed comparable results to previous studies. Obileke et al. [68] reported that the investment cost for a biogas digester was USD 1623.41. The digester provided a total methane production of 630 mL·g^−1^. Al-Wahaibi et al. [69] also used municipal solid wastes as feedstock for biogas production. The process had a break-even cost of USD 0.2944 m^−3^ and a positive net present value of USD 3108.

The current work mainly emphasized waste reusability not only for biogas and electricity production but also in the fabrication of digesters, which not only reduces the production cost but also has the potential for waste resource reclamation. For hilly areas, pine needles are one of the most prominent forest litter, accumulated in abundance. In addition, a higher lignin content makes the degradation of pine needles difficult and hence pine needles remain in the environment for longer periods. A combination of cow dung and pine needles in anaerobic digestion provided higher energy output in comparison to cow dung and pine needles alone. Both batch and continuous digestion revealed a maximum voltage output in the presence of 5 mM powdered graphite followed by 15 mM activated charcoal and 25 mM biochar. The maximum voltages observed in the presence of graphite powder were 1.5-fold and 1.3-fold higher than the control (in the absence of graphite). This work emphasized the utilization of waste for green energy generation along with the recycling of waste resources like plastic containers, graphite rods, and metal wires (from exhausted batteries) discarded from household and industrial processes.

## 5. Conclusions

The current investigation focused on the effect of conductive materials such as graphite powder, activated charcoal powder, and biochar powder on the AD of lignin-rich pine needles. The results revealed that a conductive material enhanced the AD of pine needles, which was investigated in terms of current production and methane production in batch and continuous AD processes. The time duration for the maximum current production and methane production was also shortened by the conductive materials for the AD of pine needles. In addition, the fabrication of the system included waste residues from different sources like graphite rods, etc., suggesting the recycling and reutilization of waste, which can improve the process economics and contribute to a circular economy. Large-scale trials are also needed with such an approach for the commercialization of the process.

## Figures and Tables

**Table 1:** Comparative analysis of existing biogas and electricity generation technologies.

Technology	Advantages	Limitations	References
Anaerobic digestion	Renewable energy production Waste management Carbon neutrality Substrate flexibility By-product utilization	High initial investment Complex operation and maintenance Digestion efficiency and stability Digestate management	[2,3]
Combined Heat and Power (CHP)	CHP systems are highly efficient Energy savings CHP systems contribute to greenhouse Gas emission reduction Fuel flexibility	High initial investment Complexities in system sizing, control, and operation Maintenance requirements Heat demand matching	[4]
Gasification	Gasification can utilize a wide range of feedstocks High energy conversion efficiencies Cleaner fuel production Potential for carbon capture and utilization	Complexity and cost Feedstock quality and handling Gas cleanup and tar management Scale and integration challenges	[5]
Microbial fuel cells	The generation of renewable electricity from a wide range of organic substrates. MFCs can simultaneously treat organic waste while generating electricity. MFCs have relatively low operating costs compared to traditional wastewater treatment technologies. MFCs can facilitate the recovery of valuable resources from organic waste, such as nutrients and metals.	MFCs typically exhibit lower power densities compared to other renewable energy technologies. Slow start-up and response times MFC systems can be complex to design, construct, and maintain, requiring careful attention to electrode materials, microbial inoculation, and system optimization. Limited substrate utilization MFC performance is influenced by environmental factors such as temperature, pH, and salinity, which can impact microbial activity and electrode kinetics	[6]

**Table 2:** System specifications (batch experiment).

Setup	Substrate	Composition
Control	Cow dung slurry only (CDS)	1:1, 1:2, 2:3
Test 1	Pine needle (PN) + CDS	CDS + 1% PN
Test 2	PN + CDS + Conducting material	CDS + 1% PN + Conducting material (0–30 mM)

**Table 3:** System specifications for scale-up (3 L working volume).

Setup	Conducting Material	Substrate
Control	No conductive material	Cow dung slurry only
Test 1	No conductive material	1% Pine needle + cow dung slurry
Test 2	Conductive material	1% Pine needle + cow dung slurry + Graphite powder 5 mM

## Data Availability

The data are available with the corresponding author and can be made available upon request.

## References

[1] Guo D., Li Q., Liu P., Shi X., Yu J. (2023). Power Shortage and Firm Performance: Evidence from a Chinese City Power Shortage Index. Energy Econ..

[2] Pramanik S.K., Suja F.B., Zain S.M., Pramanik B.K. (2019). The Anaerobic Digestion Process of Biogas Production from Food Waste: Prospects and Constraints. Bioresour. Technol. Rep..

[3] Kaur M., Menon V., Kumar A., Prasad B., Singh B., Sharma S., Gupta S., Mishra A.K., Hussain C.M. (2024). 6—Aerobic and Anaerobic Degradation of Bioplastics. Bioplastics for Sustainability.

[4] Hosseini S.E., Wahid M.A. (2014). Development of Biogas Combustion in Combined Heat and Power Generation. Renew. Sustain. Energy Rev..

[5] Hameed Z., Aslam M., Khan Z., Maqsood K., Atabani A.E., Ghauri M., Khurram M.S., Rehan M., Nizami A.-S. (2021). Gasification of Municipal Solid Waste Blends with Biomass for Energy Production and Resources Recovery: Current Status, Hybrid Technologies and Innovative Prospects. Renew. Sustain. Energy Rev..

[6] Hoang A.T., Nižetić S., Ng K.H., Papadopoulos A.M., Le A.T., Kumar S., Hadiyanto H., Pham V.V. (2022). Microbial Fuel Cells for Bioelectricity Production from Waste as Sustainable Prospect of Future Energy Sector. Chemosphere.

[7] Potter M.C. (1911). Electrical Effects Accompanying the Decomposition of Organic Compounds. Proc. R. Soc. B.

[8] Martinez R.D.R., Bermudez M.E.A. (2023). Production of Electrical Energy from Living Plants in Microbial Fuel Cells. Clean Energy.

[9] Schröder U. (2011). Discover the Possibilities: Microbial Bioelectrochemical Systems and the Revival of a 100-Year-Old Discovery. J. Solid State Electrochem..

[10] Gahlot P., Ahmed B., Tiwari S.B., Aryal N., Khursheed A., Kazmi A.A., Tyagi V.K. (2020). Conductive Material Engineered Direct Interspecies Electron Transfer (DIET) in Anaerobic Digestion: Mechanism and Application. Environ. Technol. Innov..

[11] Naina Mohamed S., Ajit Hiraman P., Muthukumar K., Jayabalan T. (2020). Bioelectricity Production from Kitchen Wastewater Using Microbial Fuel Cell with Photosynthetic Algal Cathode. Bioresour. Technol..

[12] Feng F., Wu C.-H., Li F., Wang X., Zhu J., Zhang R., Chen S.-C. (2024). Research on the Integration of Microbial Fuel Cells with Conventional Wastewater Treatment Technology: Advantages of Anaerobic Fermentation. Energy Convers. Manag. X.

[13] Pham T.H., Rabaey K., Aelterman P., Clauwaert P., De Schamphelaire L., Boon N., Verstraete W. (2006). Microbial Fuel Cells in Relation to Conventional Anaerobic Digestion Technology. Eng. Life Sci..

[14] Bajracharya S., Abbassi R., Yadav A.K., Khan F., Garaniya V. (2020). 14—Microbial Fuel Cell Coupled with Anaerobic Treatment Processes for Wastewater Treatment. Integrated Microbial Fuel Cells for Wastewater Treatment.

[15] Sreelekshmy B.R., Basheer R., Sivaraman S., Vasudevan V., Elias L., Shibli S.M.A. (2020). Sustainable Electric Power Generation from Live Anaerobic Digestion of Sugar Industry Effluents Using Microbial Fuel Cells. J. Mater. Chem. A.

[16] Yoshizu D., Kouzuma A., Watanabe K. (2023). Use of Microbial Fuel Cells for the Treatment of Residue Effluents Discharged from an Anaerobic Digester Treating Food Wastes. Microorganisms.

[17] Liu W., Abrha H., Dai Y., Li J., Liu M., Maryam B., Jiao S., Zhang P., Liu X. (2023). Microbial Electrolysis Cell Assisted Anaerobic Digestion System Boosted the Methane Production from Polylactic Acid by Optimizing the Methanogenesis Pathway. Biochem. Eng. J..

[18] Sondhi S., Kaur P.S., Kaur M. (2020). Techno-Economic Analysis of Bioethanol Production from Microwave Pretreated Kitchen Waste. SN Appl. Sci..

[19] El Salamony D.H., Hassouna M.S.E., Zaghloul T.I., He Z., Abdallah H.M. (2024). Bioenergy Production from Chicken Feather Waste by Anaerobic Digestion and Bioelectrochemical Systems. Microb. Cell Factories.

[20] Lovley D.R., Holmes D.E., Nevin K.P. (2004). Dissimilatory fe (iii) and mn (iv) reduction. Adv. Microb. Physiol..

[21] Geelhoed J.S., Stams A.J.M. (2011). Electricity-Assisted Biological Hydrogen Production from Acetate by Geobacter Sulfurreducens. Environ. Sci. Technol..

[22] Ahmed S.F., Mofijur M., Islam N., Parisa T.A., Rafa N., Bokhari A., Klemeš J.J., Indra Mahlia T.M. (2022). Insights into the Development of Microbial Fuel Cells for Generating Biohydrogen, Bioelectricity, and Treating Wastewater. Energy.

[23] Liu Z., Liu J., Zhang S., Su Z. (2009). Study of Operational Performance and Electrical Response on Mediator-Less Microbial Fuel Cells Fed with Carbon- and Protein-Rich Substrates. Biochem. Eng. J..

[24] Han S., Thapa K., Liu W., Westenberg D., Wang R. (2022). Enhancement of Electricity Production of Microbial Fuel Cells by Using DNA Nanostructures as Electron Mediator Carriers. ACS Sustain. Chem. Eng..

[25] Vieira S., Barros M.V., Sydney A.C.N., Piekarski C.M., de Francisco A.C., Vandenberghe L.P.d.S., Sydney E.B. (2020). Sustainability of Sugarcane Lignocellulosic Biomass Pretreatment for the Production of Bioethanol. Bioresour. Technol..

[26] Bhatia S.K., Jagtap S.S., Bedekar A.A., Bhatia R.K., Patel A.K., Pant D., Rajesh Banu J., Rao C.V., Kim Y.-G., Yang Y.-H. (2020). Recent Developments in Pretreatment Technologies on Lignocellulosic Biomass: Effect of Key Parameters, Technological Improvements, and Challenges. Bioresour. Technol..

[27] Huang L., Zeng R.J., Angelidaki I. (2008). Electricity Production from Xylose Using a Mediator-Less Microbial Fuel Cell. Bioresour. Technol..

[28] Huang C., Guo H.J., Zhang H.R., Xiong L., Li H.L., Chen X.D. (2019). A New Concept for Total Components Conversion of Lignocellulosic Biomass: A Promising Direction for Clean and Sustainable Production in Its Bio-Refinery. J. Chem. Technol. Biotechnol..

[29] Mahajan R., Nikitina A., Litti Y., Nozhevnikova A., Goel G. (2016). Autochthonous Microbial Community Associated with Pine Needle Forest Litterfall Influences Its Degradation under Natural Environmental Conditions. Environ. Monit. Assess..

[30] Singh A., Kushwaha S.P.S. (2011). Refining Logistic Regression Models for Wildlife Habitat Suitability Modeling-A Case Study with Muntjak and Goral in the Central Himalayas, India. Ecol. Model..

[31] Tiwari A., Rawat S., Adhikari R.S. (2016). Decomposition Pattern in Pinus Longifolia Leaf Litter in Chandak Forest in the Presence of Cow Dung and Urea. Int. J. Curr. Microbiol. Appl. Sci..

[32] Soong J.L., Parton W.J., Calderon F., Campbell E.E., Cotrufo M.F. (2015). A New Conceptual Model on the Fate and Controls of Fresh and Pyrolized Plant Litter Decomposition. Biogeochemistry.

[33] Sharma D.P. (2009). Biomass Distribution in Sub-Tropical Forest of Solan Foret Division (HP). Indian J. Ecol..

[34] Molina A.J., Bautista I., Lull C., del Campo A., González-Sanchis M., Lidón A. (2022). Effects of Thinning Intensity on Forest Floor and Soil Biochemical Properties in an Aleppo Pine Plantation after 13 Years: Quantity but Also Quality Matters. Forests.

[35] Chae H.M., Choi S.H., Lee S.H., Cha S., Yang K.C., Shim J.K. (2019). Effect of Litter Quality on Needle Decomposition for Four Pine Species in Korea. Forests.

[36] Safi M.J., Mishra I.M., Prasad B. (2004). Global Degradation Kinetics of Pine Needles in Air. Thermochim. Acta.

[37] Mahajan R., Nikitina A., Litti Y., Kallistova A., Nozhevnikova A., Goel G. (2019). Evaluating Anaerobic and Aerobic Digestion Strategies for Degradation of Pretreated Pine Needle Litter. Int. J. Environ. Sci. Technol..

[38] Nabi M., Liang H., Cheng L., Yang W., Gao D. (2022). A Comprehensive Review on the Use of Conductive Materials to Improve Anaerobic Digestion: Focusing on Landfill Leachate Treatment. J. Environ. Manag..

[39] Sharma D., Mahajan R., Goel G. (2019). Insights into Direct Interspecies Electron Transfer Mechanisms for Acceleration of Anaerobic Digestion of Wastes. Int. J. Environ. Sci. Technol..

[40] Madondo N.I., Rathilal S., Bakare B.F., Tetteh E.K. (2023). Application of Magnetite-Nanoparticles and Static Magnetic Field on a Microbial Fuel Cell in Anaerobic Digestion. Chem.—Asian J..

[41] Wu X., Xia A., Feng D., Huang Y., Zhu X., Zhu X., Liao Q. (2024). Intensifying Anaerobic Digestion of 5-Hydroxymethylfurfural via Granular Activated Carbon Supplementation. Int. J. Hydrogen Energy.

[42] Watson V.J., Nieto Delgado C., Logan B.E. (2013). Influence of Chemical and Physical Properties of Activated Carbon Powders on Oxygen Reduction and Microbial Fuel Cell Performance. Environ. Sci. Technol..

[43] Yellappa M., Annie Modestra J., Rami Reddy Y.V., Venkata Mohan S. (2021). Functionalized Conductive Activated Carbon-Polyaniline Composite Anode for Augmented Energy Recovery in Microbial Fuel Cells.

[44] Dong J., Wu Y., Wang C., Lu H., Li Y. (2020). Three-Dimensional Electrodes Enhance Electricity Generation and Nitrogen Removal of Microbial Fuel Cells. Bioprocess Biosyst. Eng..

[45] Ong V.Z., Wu T.Y. (2020). An Application of Ultrasonication in Lignocellulosic Biomass Valorisation into Bio-Energy and Bio-Based Products. Renew. Sustain. Energy Rev..

[46] Ahuja V., Arora A., Chauhan S., Thakur S., Jeyaseelan C., Paul D. (2023). Yeast-Mediated Biomass Valorization for Biofuel Production: A Literature Review. Fermentation.

[47] Ahuja V., Sharma C., Paul D., Dasgupta D., Saratale G.D., Banu J.R., Yang Y., Bhatia S.K. (2024). Unlocking the Power of Synergy: Cosubstrate and Coculture Fermentation for Enhanced Biomethane Production. Biomass Bioenergy.

[48] Andlar M., Belskaya H., Morzak G., Ivančić Šantek M., Rezić T., Petravić Tominac V., Šantek B. (2021). Biogas Production Systems and Upgrading Technologies: A Review. Food Technol Biotechnol.

[49] De Farias Silva C.E., Gois G.N.S.B., Abud A.K.S., Amorim N.C.S., Girotto F., Markou G., Carvalho C.M., Tonholo J., Amorim E.L., Rastegari A.A., Yadav A.N., Gupta A. (2019). Anaerobic Digestion: Biogas Production from Agro-Industrial Wastewater, Food Waste, and Biomass. Prospects of Renewable Bioprocessing in Future Energy Systems.

[50] Uddin M.M., Wright M.M. (2023). Anaerobic Digestion Fundamentals, Challenges, and Technological Advances. Phys. Sci. Rev..

[51] Wang M., Zhou J., Yuan Y.X., Dai Y.M., Li D., Li Z.D., Liu X.F., Zhang X.Y., Yan Z.Y. (2017). Methane Production Characteristics and Microbial Community Dynamics of Mono-Digestion and Co-Digestion Using Corn Stalk and Pig Manure. Int. J. Hydrogen Energy.

[52] Dang Y., Holmes D.E., Zhao Z., Woodard T.L., Zhang Y., Sun D., Wang L.Y., Nevin K.P., Lovley D.R. (2016). Enhancing Anaerobic Digestion of Complex Organic Waste with Carbon-Based Conductive Materials. Bioresour. Technol..

[53] Romero R.M., Valenzuela E.I., Cervantes F.J., Garcia-Reyes R.B., Serrano D., Alvarez L.H. (2020). Improved Methane Production from Anaerobic Digestion of Liquid and Raw Fractions of Swine Manure Effluent Using Activated Carbon. J. Water Process Eng..

[54] Zhang M., Ma Y., Ji D., Li X., Zhang J., Zang L. (2019). Synergetic Promotion of Direct Interspecies Electron Transfer for Syntrophic Metabolism of Propionate and Butyrate with Graphite Felt in Anaerobic Digestion. Bioresour. Technol..

[55] Namal O.O. (2020). Investigation of the Effects of Different Conductive Materials on the Anaerobic Digestion. Int. J. Environ. Sci. Technol..

[56] Ruan R., Cao J., Li C., Zheng D., Luo J. (2017). The Influence of Micro-Oxygen Addition on Desulfurization Performance and Microbial Communities during Waste-Activated Sludge Digestion in a Rusty Scrap Iron-Loaded Anaerobic Digester. Energies.

[57] Leng L., Yang P., Singh S., Zhuang H., Xu L., Chen W.-H., Dolfing J., Li D., Zhang Y., Zeng H. (2018). A Review on the Bioenergetics of Anaerobic Microbial Metabolism Close to the Thermodynamic Limits and Its Implications for Digestion Applications. Bioresour. Technol..

[58] Qiao W., Takayanagi K., Li Q., Shofie M., Gao F., Dong R., Li Y.-Y. (2016). Thermodynamically Enhancing Propionic Acid Degradation by Using Sulfate as an External Electron Acceptor in a Thermophilic Anaerobic Membrane Reactor. Water Res..

[59] Nevin K.P., Richter H., Covalla S.F., Johnson J.P., Woodard T.L., Orloff A.L., Jia H., Zhang M., Lovley D.R. (2008). Power Output and Columbic Efficiencies from Biofilms of Geobacter Sulfurreducens Comparable to Mixed Community Microbial Fuel Cells. Environ. Microbiol..

[60] Franks A.E., Nevin K.P. (2010). Microbial Fuel Cells, a Current Review. Energies.

[61] Lin R., Cheng J., Zhang J., Zhou J., Cen K., Murphy J.D. (2017). Boosting Biomethane Yield and Production Rate with Graphene: The Potential of Direct Interspecies Electron Transfer in Anaerobic Digestion. Bioresour. Technol..

[62] Lin R., Cheng J., Ding L., Murphy J.D. (2018). Improved Efficiency of Anaerobic Digestion through Direct Interspecies Electron Transfer at Mesophilic and Thermophilic Temperature Ranges. Chem. Eng. J..

[63] Strycharz S.M., Glaven R.H., Coppi M.V., Gannon S.M., Perpetua L.A., Liu A., Nevin K.P., Lovley D.R. (2011). Gene Expression and Deletion Analysis of Mechanisms for Electron Transfer from Electrodes to Geobacter Sulfurreducens. Bioelectrochemistry.

[64] Feng Y., Zhang Y., Chen S., Quan X. (2015). Enhanced Production of Methane from Waste Activated Sludge by the Combination of High-Solid Anaerobic Digestion and Microbial Electrolysis Cell with Iron-Graphite Electrode. Chem. Eng. J..

[65] Kardi S.N., Ibrahim N., Darzi G.N., Rashid N.A.A., Villaseñor J. (2017). Dye Removal of AR27 with Enhanced Degradation and Power Generation in a Microbial Fuel Cell Using Bioanode of Treated Clinoptilolite-Modified Graphite Felt. Environ. Sci. Pollut. Res..

[66] Zhao Z., Li Y., Yu Q., Zhang Y. (2018). Ferroferric Oxide Triggered Possible Direct Interspecies Electron Transfer between Syntrophomonas and Methanosaeta to Enhance Waste Activated Sludge Anaerobic Digestion. Bioresour. Technol..

[67] Zhao Z., Zhang Y., Woodard T.L., Nevin K.P., Lovley D.R. (2015). Enhancing Syntrophic Metabolism in Up-Flow Anaerobic Sludge Blanket Reactors with Conductive Carbon Materials. Bioresour. Technol..

[68] Obileke K., Makaka G., Nwokolo N., Meyer E.L., Mukumba P. (2022). Economic Analysis of Biogas Production via Biogas Digester Made from Composite Material. ChemEngineering.

[69] Al-Wahaibi A., Osman A.I., Al-Muhtaseb A.H., Alqaisi O., Baawain M., Fawzy S., Rooney D.W. (2020). Techno-Economic Evaluation of Biogas Production from Food Waste via Anaerobic Digestion. Sci. Rep..

